# Application of tissue engineering to the immune system: development of artificial lymph nodes

**DOI:** 10.3389/fimmu.2012.00343

**Published:** 2012-11-16

**Authors:** Tom Cupedo, Abraham Stroock, Mark Coles

**Affiliations:** ^1^Department of Hematology, Erasmus University Medical CenterRotterdam, Netherlands; ^2^School of Chemical and Biomolecular Engineering, Cornell UniversityIthaca, NY, USA; ^3^Centre for Immunology and Infection, Department of Biology and Hull York Medical School, University of YorkYork, UK

**Keywords:** lymph node, tissue engineering, stroma, bio-materials, extracellular matrix

## Abstract

The goal of tissue engineering and regenerative medicine is to develop synthetic versions of human organs for transplantation, *in vitro* toxicology testing and to understand basic mechanisms of organ function. A variety of different approaches have been utilized to replicate the microenvironments found in lymph nodes including the use of a variety of different bio-materials, culture systems, and the application of different cell types to replicate stromal networks found *in vivo*. Although no system engineered so far can fully replicate lymph node function, progress has been made in the development of microenvironments that can promote the initiation of protective immune responses. In this review we will explore the different approaches utilized to recreate lymph node microenvironments and the technical challenges required to recreate a fully functional immune system *in vitro*.

## OVERVIEW OF LYMPH NODE STRUCTURE

Immune responses in mammals are coordinated from secondary lymphoid organs (SLOs) that are positioned at strategic locations throughout the body. Naïve T and B cells continuously pass through SLO while navigating the bloodstream. Within the SLO, T and B cells scan for the presence of their cognate antigen. In the absence of antigen recognition, cells receive homeostatic survival signals allowing them to continue their journey to the next SLO. On the other hand, when T or B cells do recognize antigen, the SLOs provide an optimal environment for cellular activation, proliferation, and selection for high affinity antibodies.

To facilitate optimal lymphocyte activation, SLOs are compartmentalized into distinct cellular micro-domains that are colonized almost exclusively by either T or B cells (Crivellato et al., 2004). The generation and maintenance of B cell follicle and the T cell zone is critically dependent on cytokines, adhesion molecules, and extracellular matrix proteins made by non-hematopoietic stromal cells (Cyster, 2005; van de Pavert and Mebius, 2010). A thorough understanding of the non-hematopoietic microenvironment of SLO is therefore indispensable for lymphoid organ tissue-engineering (Mueller and Germain, 2009).

## MESENCHYMAL STROMAL CELLS

Three major subsets of functionally distinct stromal cells of mesenchymal origin are currently recognized in human and mouse lymph nodes.

### T ZONE RETICULAR CELLS

T zone reticular cells [TRC, also known as fibroblastic reticular cells (FRCs)] are the major mesenchymal stromal population in the T cell areas of the lymph nodes, located at the medullary side of the B cells follicles (Kaldjian et al., 2001; Katakai et al., 2004a; Link et al., 2007). Phenotypic, TRC are characterized by expression of podoplanin (gp38, D240) alpha-smooth muscle actin (αSMA) and the production of extracellular matrix components recognized by the antibody ERTR7 (Van Vliet et al., 1984). On a functional level, TRC secrete the homeostatic chemokines CCL21 and CCL19 which act on naïve T cells and dendritic cells (DCs), respectively (Link et al., 2007). Structurally, TRC provide a scaffold to which DC that came in through the lymph are anchored and which is used by naïve T cells to navigate the lymph node in search for DC (Bajènoff et al., 2006).

### FOLLICULAR DENDRITIC CELLS

Follicular dendritic cells (FDCs) are located within primary B cell follicles in resting lymph nodes and in the light zone of germinal centers during immune responses (Chen et al., 1978). In spite of their name, FDCs are not DCs and do not possess antigen processing machinery but rather are radio-resistant stromal cells of mesenchymal origin (Endres et al., 1999; Krautler et al., 2012). FDCs are decorated by complement receptors and Fc receptors and they use these to present unprocessed immune complexes to B cells during germinal center reactions, when B cells that have undergone somatic hypermutation test newly generated receptors for improved affinity (Kosco-Vilbois and Scheidegger, 1995). In addition, FDCs secrete the B cell chemoattractant CXCL13 that is essential to maintain the structural integrity of the B cell follicle under homeostatic conditions (Cyster et al., 2000; Allen and Cyster, 2008; Suzuki et al., 2010; Wang et al., 2011).

### MARGINAL RETICULAR CELLS

The third major mesenchymal cell type in lymph nodes are marginal reticular cells (MRCs), which are found in the outer cortex, directly underneath the subcapsular sinus (Katakai et al., 2008). MRCs have been identified in both mouse (Katakai et al., 2008) and human (T. Cupedo and M. Coles, unpublished data) lymph nodes. Phenotypically MRCs resemble so-called lymphoid tissue organizer (LTo) cells that are responsible for lymph node development in the embryo (Cupedo et al., 2004; Katakai et al., 2008; Katakai, 2012). Adult lymph node MRCs express RANKL, VCAM-1, MAdCAM-1, and podoplanin and they secrete homeostatic chemokines (Katakai et al., 2008). The physiological importance of MRCs in lymph node functioning remains in large parts to be elucidated, but these cells were shown to be involved in the shuttling of antigens from the marginal sinus to the B cell follicles (Roozendaal et al., 2009).

## ENDOTHELIAL CELLS

There are two major types of vascular network that provide nutrients, oxygen, and cellular input and output from the lymph node.

### BLOOD ENDOTHELIAL CELLS

Blood vessels enter and exit the lymph node at the medullary hilus area. In the paracortical T cell zone, the smaller venules take on a characteristic cubical appearance and are known as high endothelial venules (HEV; Miyasaka and Tanaka, 2004). These HEV are the main points of entry for naïve T and B lymphocytes. They express adhesion molecules and addressins and are decorated with stromal cell-derived chemokines. In the absence of functional HEV, recirculation of naïve lymphocytes, and thus immune surveillance, are severely impaired (von Andrian and Mempel, 2003).

### LYMPHATIC ENDOTHELIAL CELLS

Lymphatic endothelium is recognized by the characteristic expression of the receptor Lyve-1 (Banerji et al., 1999). Afferent lymphatic vessels originating in peripheral tissues carry lymph to draining lymph nodes (Oliver and Alitalo, 2005). This lymph also contains free as well as cell-bound antigens. The lymph vessels end in the subcapsular sinus, a lymphatic sinus that is located directly underneath the lymph node capsule (McMaster and Hudack, 1935; Oliver and Alitalo, 2005). From the sinus, larger antigens (>70 kDa) can be captured by lymph node-resident subcapsular macrophages or are carried by migratory antigen-presenting cells including DCs and neutrophils into the organ (Junt et al., 2007), smaller antigens can enter by diffusion through the conduit network and be captured by antigen presenting cells in the lymph node (Carroll and Isenman, 2012). Efferent lymphatic vessels originate in the lymph node hillus and allow cells to leave the organ and re-enter the blood stream or travel through the lymph to a subsequent lymph node (Forster et al., 2012).

## CAPTURING THE STRUCTURE AND COMPLEXITY OF A LYMPH NODE

**FIGURE 1:**
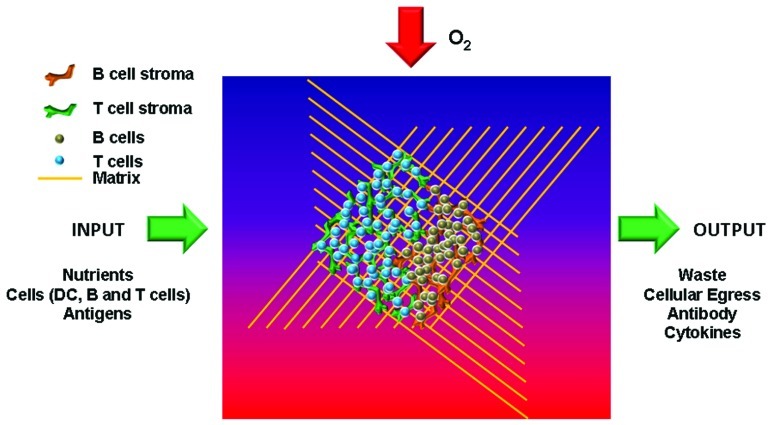
**Key features of an artificial lymph node**. To recreate functional lymph nodes *in vitro* several key constituents need to be incorporated. These include specific stromal cells for T and B areas in order to generate the typical microarchitecture. In addition, nutrients, oxygen, and antigens need to be able to enter the organoid, while functional end products such as antibodies and cytokines should be able to be extracted from the organ.

Despite their relative modest size, bio-engineering a lymph node presents a formidable challenge due to the high level of complexity resulting from the large variety of cell types, the highly organized stromal and lymphoid structure, the rapid cellular motility of lymphocytes and DCs and the staggering cell density (key features summarized in **Figure 1**). The multitude of cell types and the scale of the engineering, with complex changes in microenvironments occurring within a few microns, make engineering a lymph node more complex than the engineering of larger organs like the heart or kidneys. From immunohistological and flow cytometric measurements it can be estimated that within 1 mm^3^ of lymph node tissue approximately 1 to 2 × 10^6^ cells reside, accounting for a cell density of about one billion cells/ml (our unpublished data). Within this cell mass highly organized lymphatics, marginal reticular zone stroma, T cell zone stroma, multiple B cell follicles, vascular networks and medullary regions containing plasma cells and macrophages are present which are all highly different localized microenvironments (Crivellato et al., 2004). Even in extremely high dense tissue culture, maximum cell densities are in the range of 5 to 10 × 10^6^ cells/ml, which is two orders of magnitude less dense, indicating that there is hardly any liquid phase within the lymph node parenchyma just a compact cellular environment where cellular interactions and cell–extracellular matrix interactions dictate rapid cell migration. The lack of a liquid interface indicates that free diffusion of cytokines, chemokines, and growth factors is unlikely to occur by a simple gradient as observed *in vitro* assay systems and rather is controlled through direct contact with the stromal network that produce homeostatic chemokines and cytokines, sequestration of these factors by extracellular matrix and by the rapid motility of cytokine-secreting activated lymphocytes in the lymph node. All of these effects indicate that vast differences exist between to the biology measured in a culture dish and the biology operational *in vivo* that needs to be recapitulated in the bio-engineered lymph node.

## SOURCE OF MESENCHYME

Stromal cells provide the structural basis for both lymph node structure and function and their faithful incorporation into an *in vitro* system is thus a necessity for creation of a functional artificial lymph node environment. A prerequisite for lymph node modeling is therefore the establishment of mesenchymal cell cultures that can take on the function of lymph node stromal cells found *in vivo*. Unlike most other organs that involve layers of epithelium and fibroblasts or endothelium and muscle cells, the lymph node stroma contains an open lattice network of different types of mesenchymal stromal cells that permits rapid motility of immune cells.

There are three main practical approaches that can be utilized to generate mesenchymal cells for use in tissue engineering: (1) the application of immortal fibroblastic cell lines from a variety of different tissues (Suematsu and Watanabe, 2004; Tomei et al., 2009), (2) the use of primary stromal cells isolated from lymph nodes (Tomei et al., 2009), and (3) the differentiation of lymph node stroma from mesenchymal progenitor cells (Benezech et al., 2012; Zheng et al., 2012). Although fibroblastic cell lines from non-lymphoid tissues are easy to culture they are unlikely to mirror the gene expression pattern found in FRCs or FDCs. The culture of primary stromal cells from lymph nodes has been challenging, and this has only been successfully accomplished by a few groups (Katakai et al., 2004b; Onder et al., 2012). Prolonged culture of these cells has led to a loss of phenotype requiring supplement of the cells with exogenous cytokines and chemokines (Suto et al., 2009). Differentiation of lymphoid stromal subsets from progenitor cells would be the most ideal method to acquire lymphoid stromal cells, but reproducible protocols have not yet been developed.

## THE OXYGEN CHALLENGE

Vascular networks are both the site of cellular input and egress from lymph nodes but more importantly they provide the high levels of oxygen and nutrients required by highly motile immune cells. Any structure larger than 80–100 µm will become highly hypoxic in the absence of oxygen supply and this problem is amplified in lymph nodes, which have highly motile cells and have a cell density of one billion cells/ml (our unpublished data). One of the key engineering challenges in developing an artificial lymph node is providing an environment that can support the oxygen need of the lymph node environment. Possible scenarios include limiting the organ size, lowering the cell density, or providing artificial blood vascular networks or hollow fibers for gas diffusion and nutrient supply (Zheng et al., 2012). Artificial vascular beds can be generated through the formation of microfluidic networks in bio-compatible gels. These networks can be seeded with endothelial cells and vascular smooth muscle cells leading to the development of vascular networks that can support vascular sprouting and would lead to the incorporation of a highly structured vasculature *in vitro* (Zheng et al., 2012). Even though including functional vascular networks into engineered organs will be technically challenging, incorporation of the efficient transport of oxygen and nutrients into the organoid is essential for capturing normal lymph node function.

## TOWARD 3D CULTURE

A variety of different cellular approaches have been used by bio-engineers to recreate tissues and organs *in vitro*. These include three-dimensional (3D) culture of cells in extracellular matrix or on artificial materials (Tomei et al., 2009), the formation of spheroids (Lin and Chang, 2008), use of de-cellularized tissues (Mirsadraee et al., 2006), and cell printing (Guillotin and Guillemot, 2011). Culturing of cells in a 3D environment for tissue engineering and regenerative medicine has been performed on a number of different types of materials all with their inherent advantages and disadvantages.

Non-biological materials include sponges, synthetic polymers and peptide hydrogels, electrospun polycarbonate fibers, and synthetic multilayer surfaces.

Electrospun fibers and multilayer surfaces provide a 3D surface that permits attachment and growth of stromal cells in 3D (Holmes et al., 2012). The disadvantage of this technology is that in essence it is a 3D environment where cells grow in 2D, attached to an inert material, leading to very different interactions between stromal cells as they are not forming a 3D self-supporting network. Moreover, interactions with lymphocytes and DCs will also be fundamentally different from those observed *in vivo* as they will interact only with non-adhered surface of stromal cells. Alternative synthetic matrices include sponges and polymer hydrogels (Tibbitt and Anseth, 2009). Depending on the pore size, sponges are essentially like electrospun polycarbonate, providing a mere artificial substrate for cell growth. Polymer hydrogels form into solid gels through the exclusion of water and condensation of polymer, leading to the encapsulation of cells into the hydrogel rather than the formation of 3D networks. These types of gels work extremely well for cell types such as chondrocytes but are likely to be of limited application for engineering lymph node environments.

Biological materials including agarose, laminin, Matrigel, fibrin, and collagen gels are all commonly used in tissue engineering. Agarose is an inexpensive material that is commonly used to culture hematopoietic colonies. Its advantages are the ability to be formed into a variety of different tensile strengths and agarose gels can be used to induce a certain level of cellular organization. A major disadvantage of agarose is the fact that it cannot be biologically restructured during culture, this restricts the mobility of cells and it is technically challenging to extract cells for analysis, limiting its application. Matrigel is a mix of laminin, fibronectin, and collagen of an unknown constituency and is commonly used in tissue engineering and tumor metastasis assays (Hughes et al., 2010). Matrigel is secreted by a mouse tumor cell line and probably represents a type of extracellular matrix normally deposited in tumor microenvironments and surrounding blood vasculature. Despite its popularity, Matrigel cannot be easily remodeled by cells, and migration through Matrigel usually involves digestion of the matrix by matrix metalloproteinase proteins (MMPs), rather then active squeezing and crawling of cells through the matrix (Lammermann et al., 2008). It is also very different in constituency to the type of extracellular matrix found in lymph nodes. Collagen gels are commonly used in a variety of tissue engineering applications and different types of collagens are important components of extracellular matrix in a number of organs including lymph nodes. Collagen I bundles form the flexible outer capsule of the lymph node and the conduits that connect lymphatics to the HEVs providing structural support for the stromal cell network and channels for movement of factors and low molecular weight antigens into the parenchyma of the lymph node (Lammermann et al., 2008). Collagen IV is secreted by TRC and forms a layer of extracellular matrix around the these cells (Sixt et al., 2005). To date, there is no evidence that this matrix deposition is important to the structural integrity of the lymph node. Collagen is a highly bio-compatible material and has been applied to a number of different tissue engineering situations but is easily contracted by cells when they apply force to the collagen gel and is more challenging to work with due to its relative low tensile strength in comparison to other biomaterials (Cross et al., 2010).

Alternative tissue engineering approaches used to form 3D organ-like cultures include spheroids which have been successfully utilized to study bone biology, endothelial cell outgrowths, and the formation of breast and prostate tissues (Villadsen et al., 2007). Spheroids are formed through the culture of cells in methyl cellulose in hanging droplets, inducing the formation of a compact tissue that can take on a higher degree of organization reminiscent of the *in vivo* organ. Although this approach has worked well for a variety of different tissues, the technology has to date not been utilized for the formation of artificial lymph node microenvironments. The main reason being that spheroids form into a very compact cell aggregate which is fundamentally different from the open lattice stromal cell network found in LNs.

Cell printing and encapsulation technology have been used to form vascular networks and 3D bone structures (Fedorovich et al., 2011). However, this technology lacks the resolution to be of application when generating a complex structure like a lymph node.

Capturing the complex structure of lymph nodes with separate B and T cells zones is challenging. Most work to date has not tried to impose structure upon the bio-engineered tissues rather it has relied upon self-organization. In developing lymph node B cells form into B cell follicles guided by a complex system of stromal-derived chemokines (Mebius, 2003). Recapitulating the appropriate signal *in vitro* will be essential for achieving organized artificial lymphoid tissues.

## CURRENT *IN VITRO* MODELS OF LYMPH NODE MICROENVIRONMENTS

To recreate the lymph node environment a number of different approaches have been utilized, and we will highlight several of those here. For a more comprehensive overview of the existing models see Kobayashi et al. (2011).

### COLLAGEN/POLYURETHANE MATRIX

By combining type I collagen with polyurethane a composite matrix was generated that provided sufficient tensile strength to counteract the contraction induced by the stromal cells seeded in the matrix. Using immortalized TRC lines, it was convincingly shown that these cells not only behave totally different in 3D compared to 2D, but most importantly that culturing the matrix under flow induced structural remodeling and production of chemokines (Tomei et al., 2009).

### BIOREACTOR

In order to recreate immunological interactions rather then to faithfully rebuild the lymph node structure, progress has been made using membrane-based perfusion systems that can be seeded with several types of matrices. After loading the bioreactor with DCs and lymphocytes the production of inflammatory cyto-kines as well as tissue architecture could be assessed (Giese et al., 2006, 2010).

### COLLAGEN SPONGE

The best results in terms of immune responses have been achieved using collagen sponges, seeded with stromal cells and DC and subsequently transplanted in mice. Even though these sponges attract and organize B cells, form germinal centers and lead to plasma cell differentiation in a very efficient manner, they fall beyond the scope of this review as most of the these features are achieved *in vivo *(Suematsu and Watanabe, 2004; Okamoto et al., 2007).

## FUTURE DIRECTIONS AND CHALLENGES

A number of factors have limited the capacity to generate artificial lymph nodes these include the size, the development of hypoxia in larger tissues, and the engineering complexity of the lymph node. One of the important engineering challenges will be to recreate a compact environment *in vitro* complete with stromal networks and to maintain cell motility, behavior, and function. This will require developing tissue engineering on an immensely small scale, the development of techniques to form stromal networks from stem cell precursors, and methods to culture cells in very high densities. So far a number of groups have shown that this can be done *in vivo* and some of the complexity has been modeled *in vitro* using custom-designed bioreactors. Advancement in bioreactor development and the culture of stromal cells provides a platform for advancements in this bioengineering challenge.

## CONCLUSION

Over the last 15 years bio-engineers have developed novel artificial organs that have started to appear in the clinic, one of the great challenges left is to successfully develop an artificial immune system that can reproduce complex immune responses *in vitro*. Through designing and engineering artificial lymph nodes immunologists will develop a tool that could be used to dissect the molecular and biophysical mechanisms controlling human immune responses in health and disease.

## Conflict of Interest Statement

The authors declare that the research was conducted in the absence of any commercial or financial relationships that could be construed as a potential conflict of interest.
